# LOX-1 and cancer: an indissoluble liaison

**DOI:** 10.1038/s41417-020-00279-0

**Published:** 2021-01-05

**Authors:** M. Murdocca, C. De Masi, S. Pucci, R. Mango, G. Novelli, C. Di Natale, F. Sangiuolo

**Affiliations:** 1 Department of Biomedicine and Prevention, Tor Vergata University, Rome, Italy; 2 Cardiology Unit, Department of Emergency and Critical Care, Tor Vergata Hospital, Rome, Italy; 3 Department of Electronic Engineering, Tor Vergata University, Rome, Italy

**Keywords:** Cancer therapy, Tumour biomarkers

## Abstract

Recently, a strong correlation between metabolic disorders, tumor onset, and progression has been demonstrated, directing new therapeutic strategies on metabolic targets. *OLR1* gene encodes the LOX-1 receptor protein, responsible for the recognition, binding, and internalization of ox-LDL. In the past, several studied, aimed to clarify the role of LOX-1 receptor in atherosclerosis, shed light on its role in the stimulation of the expression of adhesion molecules, pro-inflammatory signaling pathways, and pro-angiogenic proteins, including NF-kB and VEGF, in vascular endothelial cells and macrophages. In recent years, LOX-1 upregulation in different tumors evidenced its involvement in cancer onset, progression and metastasis. In this review, we outline the role of LOX-1 in tumor spreading and metastasis, evidencing its function in VEGF induction, HIF-1alpha activation, and MMP-9/MMP-2 expression, pushing up the neoangiogenic and the epithelial–mesenchymal transition process in glioblastoma, osteosarcoma prostate, colon, breast, lung, and pancreatic tumors. Moreover, our studies contributed to evidence its role in interacting with WNT/APC/β-catenin axis, highlighting new pathways in sporadic colon cancer onset. The application of volatilome analysis in high expressing LOX-1 tumor-bearing mice correlates with the tumor evolution, suggesting a closed link between LOX-1 upregulation and metabolic changes in individual volatile compounds and thus providing a viable method for a simple, non-invasive alternative monitoring of tumor progression. These findings underline the role of LOX-1 as regulator of tumor progression, migration, invasion, metastasis formation, and tumor-related neo-angiogenesis, proposing this receptor as a promising therapeutic target and thus enhancing current antineoplastic strategies.

## Introduction

Cancer is a leading cause of death worldwide with continuance and increasing incidence in the 21st century. The situation is so alarming that every fourth person is having a lifetime risk of cancer [1].

In the current era of precision medicine, research is almost concentrated on providing treatment that is more effective by focusing on patient-specific factors. This is particularly important in heterogeneous cancers where the frequency and mortality remain both troublingly high.

With the recent progress in understanding the molecular mechanisms underlying cancer development, dissemination, resistance to chemotherapy, and radiation therapy, it is now easier to select the most proper strategy for managing cancer.

The goal of future research is to identify those biomarkers that could allow a non-invasive and cost-effective diagnosis, as well as to recognize the best prognostic panel of biomarkers and then define personalized treatments.

Among these biomarkers, the lectine like oxidized low density lipoprotein receptor-1 (LOX-1) has been recently reported as playing a pivotal role for ox-LDL receptor and the related downstream signaling pathways in the onset, progression, and metastasis of cancer [2].

LOX-1 is canonically known as the receptor of oxidized low-density lipoprotein (ox-LDL). It has been extensively studied in the context of atherosclerosis and associated vascular diseases, including hypertension and stroke. As demonstrated by in vivo studies, LOX-1 plays an important role in proatherogenic processes. Specifically, it is involved in macrophage foam cell and plaque formation, endothelial dysfunction, proliferation of vascular smooth muscle cells, platelets aggregation, and leukocyte recruitment [3].

In this review, we outline the function exerted by LOX-1 in tumorigenesis, insurgence, and progression of different human cancers and thus new diagnostic potentialities that this marker plays in the early detection of this disease (Table 1).

**Table 1:** The involvement of LOX-1 in different kinds of cancer.

Cancer	Tumor database	Blood samples	Histological samples	Cells	Animals	LOX-1 expression levels	LOX-1 role	Ref.
Colorectal		238	28–100	DLD-1, HCT-8, RKO	10 CD-1 nude mice	Overexpression correlates with tumor stage and grade	Prognostic	[29–31]
Breast			47	MCF-10A ER-Src and MCF-10A pBABE MCF-12F, MCF-7, MDA-MB231, SKBR-3.	15 nu/nu nude mice	Overexpression correlates with tumor grade	Predictive	[51–54]
Prostate	256 primary prostate tumors	75		LNCaP and PC-3 and human C4-2 cell lines	BALB/c mice	High expression in more aggressive and metastatic stage of cancer	Prognostic	[23–28]
Squamous NSCLC			13			High expression	Prognostic	[60–62]
Lung adenocarcinoma		19		HPAEpic, HCC827, A54 9, H441, H446, H460, H5 22	BALB/C nude mice	High expression	Prognostic	[60, 61]
Gastric	100 gastric cancer cases		80	MGC80-3 and AGS		Overexpression	Prognostic	[63–67]
Glioblastoma		15	23			Lox1pmn +	Prognostic	[72, 73]
Osteosarcoma			61	U2-OS, SAOS-2, 143b and MG63	8 BALB/C nude mice	Overexpression	Prognostic	[74, 75]

The knowledge we are gaining from the activities carried out by this receptor is opening new exciting possibilities for the early diagnosis and treatment of cancer.

Importantly, LOX-1 inhibition could represent a promising strategy in the treatment of a peculiar subgroup of tumors overexpressing this “chameleonic” receptor, possibly leading to the development of more effective complementary therapies against cancer.

## The lectin-like oxidized low-density lipoprotein receptor-1 (LOX-1)

In *Homo Sapiens* LOX-1 is a homodimer of 52 kDa expressed by macrophages, endothelial cells, smooth muscle cells, fibroblasts, platelets, and neurons [3]. It belongs to class E scavenger receptors (SR) and contains a domain common to C-type lectin receptor (CLRs). LOX-1 protein includes four domains: a short N-terminal cytoplasmic domain, a transmembrane domain, a neck domain, and a lectin-like extracellular C-terminal domain (CTLD). The latter, after combining with ox-LDL, forms a disulfide-linked heart-shaped homodimer and creates larger functional oligomers through non-covalent interaction [4, 5].

Caveolae/lipid rafts include LOX-1 receptors within plasma membranes. A spatial disorganization and a marked loss of function appear when LOX-1 is chronically exposed to statins [5], representing competitive inhibitors of the cholesterol synthesis enzyme 3-Hydroxy-3-Methylglutaryl-CoAReductase (HMGCR). Specifically, if employed as an adjuvant to chemotherapy, statins can trigger chemo sensitivity in cancer cells, by interfering LOX-1-mediated recognition of ox-LDL [6].

*OLR1* gene (OMIM#602601) codifies for LOX-1 receptor. It is mapped on the p12.3-p13.2 region of human chromosome 12 [7] and contains six exons and five introns. *OLR1* gene is 7000 bp long and can produce three splice variants: the full-length mature *OLR1* (NM_002543), functionally active in binding and internalizing ox-LDL into plasma membrane, mainly in the endothelial cells. The second one, spanning 950 bp, is named transcript variant 2 (NM_001172632) or *OLR* Δ4. It lacks exon 4 and thus a part of the ligand recognition. Finally, the third transcript variant (NM_001172633) is 1008 bp long, without exon 5, due to a premature stop codon and a consequent termination. The protein encoded is called LOXIN, missing two-thirds of the CTLD and thus compromising the ox-LDL binding activity [8]. LOXIN is referred as playing an important protective role against mature LOX-1 [9].

LOX-1 is mostly upregulated by ox-LDL and also by pro-inflammatory cytokines, angiotensin II, and endothelin [10, 11]. It needs interactions with protein for intracellular signaling, because it has no known enzymatic or catalytic activity in its cytoplasmic tail [12]. The receptor, by inducing ox-LDL uptake, lipolysis, foam cell generation, and last atheroma plaque formation, represents a major driver of atherosclerosis development [13]. Moreover, also a soluble form of LOX-1, due to a proteolytic cleavage in its membrane-proximal extracellular domain, could exist and it is upregulated in patients with acute coronary syndromes [14].

## LOX-1, metabolism and cancer

**Fig. 1:**
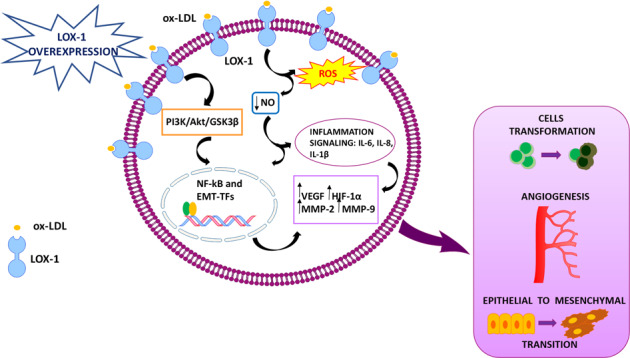
Intracellular molecular mechanisms leading to metabolic reprogramming and cell transformation, mediated by LOX-1 overexpression. Ox-LDL binding to LOX-1 increases ROS formation and NO release reduction, alternatively it can activate the PI3K/AKT/GSK3β cascade. The activation of both pathways results in the triggering of transcription factors associated to epithelial to mesenchymal transition (EMT-TFs) and of NF-kB. The NO release reduction can also activate the inflammatory signaling (IL-6, IL-8, and IL-1β). The final result is the activation of hypoxia pathways (VEGF, HIF-1α) and the enhancement of mesenchymal markers expression (MMP-2 and MMP-9). The outcome of all these processes determines cell transformation, angiogenesis, and the epithelial to mesenchymal transition.

Epidemiological studies report that one of the main features of several tumors is represented, as a primary event, by the activation of lipid metabolism. The molecular mechanisms leading to activation of metabolic reprogramming and cellular transformation are described in Fig. 1. Many data underline a strong association among obesity, metabolic syndrome, insulin resistance, inflammation, and increased cancer risk [15, 16]. Reactive oxygen species (ROS) are upregulated by the binding of ox-LDL to LOX-1 [17]. ROS causes the oxidative DNA damage, in vivo oxidation of lipids and proteins. A link between increased levels of free radicals, lipid peroxides, and carcinogenesis has been reported by several studies [18, 19]. In endothelial cells, after ox-LDL binding to LOX-1, nitric oxide (NO) release results to be decreased while nuclear factor kappa B (NF-kB) is evidently activated [20, 21].

LOX-1 role in cancer susceptibility has not been addressed yet. Cancer cell-specific transcriptional signature revealed several common genes related to metabolic disorders, inflammation, and carcinogenesis, highlighting the relevance of lipid metabolism in cellular transformation [22]. Specifically, LOX-1 is upregulated in 57% of bladder and cervix cancer cells, 11% of mammary gland cancer cells, 10% of lung cancer cells, and in 20% of colorectal cancer (CRC) cells, as reported in the meta-analysis of gene expression profiles of about 950 cancer cell lines [22].

## LOX-1 and prostate cancer

Prostate cancer (PCa) is the second most frequent cancer in men and the fifth leading cause of death worldwide. About 1,276,106 new cases of prostate cancer were reported worldwide in 2018, with higher prevalence in the developed countries [23]. PCa could be asymptomatic at the early stage and often has an indolent course that may require only active surveillance [23]. Treatment options consist of surgery, radiation therapy, but also hormone therapy. PCa is androgen-dependent; this is why until 2004 androgen deprivation therapy was associated to a secondary hormonal manipulation, especially for metastatic disease. In the last decade, six systemic agents have been approved for the treatment of castration-resistant prostate cancer [24].

Concerning the discovery of prostate cancer markers, clinical and epidemiological data suggested that coronary artery disease shares etiological pathways with this tumor [25]. High levels of *OLR1* expression are associated with more aggressive and metastatic stage, like stages III and IV, and lymph node metastasis [25]. A study conducted by Wan et al. established a relation between ox-LDL concentrations and prostate cancer, starting from the assumption that oxidative stress is one of the main events characterizing the most aggressive tumor phenotypes [25, 26]. Their research has found out not only elevated levels of ox-LDL in primary and metastatic PCa, but also an increased proliferation, migration, and invasion rate of prostate cancer cells, mediated by ox-LDL through *OLR1*. These results make ox-LDL a hallmark of PCa progression and prognosis. Moreover, other studies placed the aim to clarify the role of *OLR1* in prostate cancer [25]. Chavarrìa et al. deepened the contribution of this receptor in PCa angiogenetic process, using a xenograft model in which an increment of tumor vascularization was observed [27]. They used C4-2 prostate cancer cells in where LOX-1 transcript was overexpressed or alternatively knocked down. The first situation included the transduction of cells by a lentiviral expression vector encoding *OLR1* gene, the second one provided short hairpin RNA (shRNA) against *OLR1* mRNA. The cell lines obtained were incubated with ox-LDL and then the expression of the pro-angiogenic markers (VEGF, matrix metalloproteinase: MMP-2 and MMP-9) was evaluated, observing that this expression was reduced or incremented in cells knocked down or overexpressing *OLR1* transcript, respectively [27]. Thus, the hypothesis regarding the possible promotion of angiogenesis by ox-LDL-mediated-LOX-1 activation was clearly confirmed [27].

Another aspect of LOX-1 involvement in PCa is represented by the enhancement of the epithelial to mesenchymal transition (EMT), through a lowered expression of epithelial markers (E-cadherin and plakoglobin) and an increased expression of mesenchymal ones (vimentin, N-cadherin, snail, slug, MMP-2, and MMP-9) [28]. It has been demonstrated that LOX-1 increases the tumorigenic potential of prostate cancer cells, promoting their invasion and migration, and that it constitutes a fundamental factor for tumor growth in nude mice.

All these observations make LOX-1 a prognostic and diagnostic factor of prostate cancer. Moreover, the action performed by ox-LDL through LOX-1 could also correlate clinical aspects of obesity to prostate cancer. This association also explains why obese patients with prostate cancer have an accelerated tumor progression and a greater probability of developing metastasis in comparison with normal weight patients [28].

## LOX-1 and colorectal cancer

According to the last statistics of the International Agency for Research on Cancer (IARC) of the World Health Organization (WHO), Colorectal cancer is the third most frequent malignant disease around the world (1.85 million new cases/year; 10.2% of total malignancies) [29]. It is one of the most widely studied cancers worldwide, conferring significant morbidity, mortality, and costs to the public health system. Colorectal cancer may be unluckily silent for a long time in a large number of patients: recurrence and metastases are the primary causes of mortality. Twenty-five percent of patients with CRC have metastatic onset disease and up to 35% of these individuals will later develop local or remote recurrence. Surgery is an essential treatment in non-metastatic forms from both the colon and rectum.

Our first study on the involvement of LOX-1 in CRC has evidenced that LOX-1 resulted to be strongly increased in the 72% of human colon carcinomas, and strongly overexpressed in 90% of highly aggressive and metastatic tumors, if compared to healthy counterpart of the same patients [30]. Moreover, its expression was directly correlated to tumor stage and grade. LOX-1 mRNA in vitro reduction strongly affects the maintenance of transformed state, growth and tumorigenicity in two colon cancer cell lines, DLD-1 and HCT-8. When LOX-1 mRNA was inhibited by short hairpin RNA interference (shRNAi) or by neutralizing antibodies, cell proliferation rate was significantly lowered. Similarly, the wound-healing assay revealed an evident impairment in closing the scratch.

Following in vivo research has been focused on the role of LOX-1 in colon tumorigenesis by xenografting procedures [31]. High-grade human metastatic colorectal cancer cells, downregulated for LOX-1 by shRNAs, have been injected into Nude mice, both subcutaneously and intravenously. LOX-1 inhibition acts on tumor engraftment and metastasis development by impacting tumor angiogenesis. In addition, metastasis formation is strongly prevented by inducing a downregulation of VEGF‐A165, HIF‐1α, and β‐catenin, whose expression is involved in cell migration and metastasis [31].

A recent research reports high levels of LOX-1 in a serum sample of 238 CRC patients and in 100 tissue samples [32]. High serum levels of LOX-1 determine a poorer overall survival of patients with respect to those presenting low levels, as well as their prognosis. In fact, serum LOX-1 represents an independent prognostic factor in multivariate analysis for overall survival in liquid biopsy. In addition, inflammatory factors such as white blood cell count, C-reactive protein level, neutrophil/lymphocyte ratio, and monocyte/lymphocyte ratio are significantly higher in the group with high serum LOX-1 levels.

All these data corroborate the contribution of LOX-1 for inhibiting tumor progression and metastasis, integrating in this way the therapeutic strategies against colorectal cancer. LOX-1 can be considered a regulator of tumor progression, migration, invasion, metastasis formation, and tumor-related neo-angiogenesis.

If cancerous tissues are not available, its detection in liquid biopsy can support in a significant manner both cancer diagnosis and treatment.

## LOX-1, volatile organic compounds and colorectal cancer

Metabolomics and metabonomics became widely diffused disciplines that study the interplay of metabolites and their measurement for diagnostic purposes [33]. The importance of these studies is based on the evidence that chemical signals captured in body secretions and excretions contain information about the cellular processes and ultimately in the health status [34].

The volatile fraction of the metabolome, the volatolome, is gaining a growing interest because of a supposed simplicity of sample collection, the intrinsic non-invasiveness of measurements and the wide availability of analytical methods. Studies evidenced that patterns of volatile organic compounds (VOCs) has been shown to be related to a vast range of phenomena observable in vitro, even at single-cell level [35] and in vivo [36].

Several instrumental techniques are available for the analysis of volatolome composition, including gas chromatograph and mass spectrometers. On the other hand, portable and easy to use instruments based on sensors arrays (so-called electronic noses) are also becoming available. Electronic noses have been demonstrated to be sufficiently sensitive and selective to identify diseases analyzing various human samples such as breath [37], urine [38], and sweat [39].

Breath and urines are likely the largest collector of the volatolome. This prevalence is due to the chemical and physical mechanisms that leads from the excretion of metabolites from cells and tissues, to their dilution in blood. The exchange at the blood–air interface in the lungs and the renal functions in kidneys, regulate the passage of metabolites in breath and urines.

Breath analysis demonstrated to provide the detection of cancers affecting, besides lungs, disparate organs such as breast, colon, and prostate and, obviously, lungs [40]. Similarly, the analysis of volatile metabolites in urines provided the discrimination of bladder, prostate, lung, colorectal, breast, and oesophageal cancers [41].

Although the growing number of experimental proofs, the origin of the involved VOCs is still obscure. The most plausible causes include oxidative stress, gene mutations, and the Warburg effect [42]. In this regard, the influence of LOX-1 in promoting the oxidative stress and the production of reactive oxygen species is well-known [30]. Thus, it is reasonable to suppose that the knockdown of LOX-1 in cancer cells should influence not only cell proliferation, but also the related production of volatile compounds. This hypothesis has been investigated both in vitro and in vivo in colorectal cancer [30, 31], The in vitro study compared the VOC emission from cultures of DLD-1 metastatic colon cancer cells in which LOX-1 has been downregulated and scramble DLD-1 cells. The volatile compounds released by cell cultures were sampled with a solid-phase micro-extraction (SPME) and analyzed by a gas chromatography mass spectrometer. Results show a modest release of VOCs: only seven compounds were recursively found in at least three samples. These compounds are putative products of oxidative stress and their anomalous concentrations have been observed in various cancers [43, 44]. Specifically, a butyrate derivative is affected by the LOX-1 knockdown. Noteworthy, the concentration of the butyric derivative decreases with the culture days and it is exclusively found in scramble cells, in which LOX-1 has not been inhibited.

**Fig. 2:**
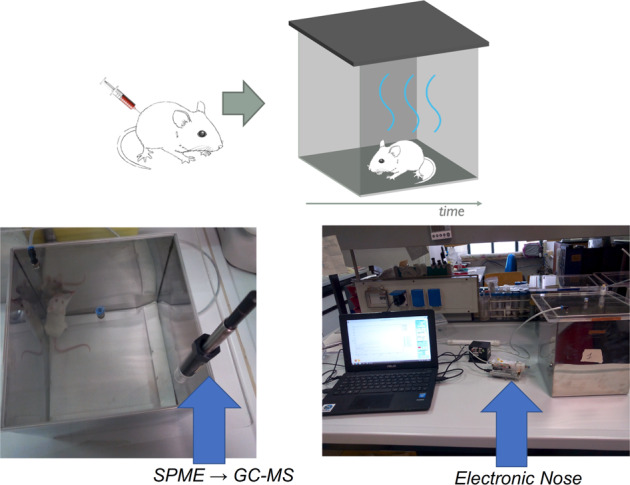
experimental setup for the volatolome measurement of xenografted mice. Each individual animal was kept in a cage during the measurement. The volatile compounds were sampled with an SPME fiber for the GC-MS analysis. Electronic nose measurements were performed conveying with a pump the air from the cage to the sensors.

The effect of LOX-1 downregulation was further investigated in vivo in mouse xenograft model [31] by volatile compounds analysis. Mice volatolome were measured with gas chromatography and with an electronic nose. The adopted experimental setup, shown in Fig. 2, enabled the measurement of the total volatolome of each animal. Mice volatilome results to be totally different respect to the in vitro experiment; rather, the VOCs found in mice are common volatolome elements. Mice were injected both subcutaneously and endovenously. The method of injection strongly affect the quality and the quantity of the volatile compounds. In endovenously injected mice a largest VOCs abundance in scramble and LOX-1_RNAi_ mice are reported. The evolution of volatile compounds was confirmed by electronic nose measurements.

Thus, the analysis of VOCs offers an independent observation of the role played by LOX-1 in tumor progression, and it is confirmed as a viable instrumental alternative to follow the progression of tumors.

## LOX-1 and breast cancer

Breast cancer is the main occurring cancer in woman worldwide and the second most common cancer overall. It represents the main cancer cause of death among women: over 2 million new cases in 2018 [45] (https://www.who.int/cancer/prevention/diagnosis-screening/breast-cancer/en/). To date, the treatment of this disease involves the integration of different options based on surgery, radiotherapy, and systemic therapy. The choice of the most appropriate therapeutic approach must consider cancer stage and grade, but also its localization and molecular characteristics at the time of the diagnosis [46].

Actually, it is well-known the correlation between metabolic disorders and carcinogenesis. Different evidences have highlighted the link between the intake of fat-enriched diet, the development of lipid-related diseases, and the increased incidence of breast cancer.

The altered metabolism makes transformed cells able to oxidize fatty acid at much higher rates than their non-tumor counterpart [47, 48]. Recently, Wang et al. have deepened this aspect of the disease, focusing the attention on the so-called metabolic reprogramming in triple-negative breast cancer (TNBC), which is one of the most aggressive tumors [49]. They disclosed tumor cells lipid metabolism, where one aspect is embodied by triglycerides in circulating lipoprotein particles that can provide an additional exogenous source of fatty acid. Therefore, TNBC exhibits enhanced lipid uptake, hydrolyzing triglycerides into fatty acids thanks to the secretion of lipoprotein lipase and expressing CD36 [49].

Risen levels of serum ox-LDL have been detected in patients with breast cancer [49]. Moreover, its receptor LOX-1 is overexpressed in 70% of human breast tumor tissues, correlating positively with cancer grade and stage [50]. Furthermore, *OLR1* is overexpressed in a coordinated way with other lipid metabolic genes (*GLRX* and *SNAP23*) in late-stage breast cancer tissues [51].

siRNA-mediated inhibition of *OLR1* expression suppresses the growth of breast cancer cells and reduces the tumorigenicity, highlighting its importance for the maintenance of transformed state [51].

Already in 2007, a study has reported the stimulated expression of LOX-1 driven by tumor necrosis factor alpha (TNF-α) in a dose and time-dependent manner [52]. This cytokine is known to be a potent pro-metastatic mediator and it leads to breast cancer cells-endothelial cells interactions, through a regulation action exerted by LOX-1 [52]. Furthermore, it was also demonstrated the contribution of the oncogene TBC1D3 in the migration of human breast cancer cells upregulating *OLR1*, through the control of TNFα/NF-κB signaling [53].

Interestingly LOX-1 can help to establish a correlation between obesity and breast cancer malignancy. Indeed, obesity is associated with increased adipose tissue hypoxia and this defines a condition, which can assess a pro-malignancy environment in breast tissue, particularly linked to the loss of estrogen receptor (ER). This hypothesis has been supported by the coculture of adipocytes with ER-positive breast cancer cells. This experimental condition induces the overexpression of HIF-1α, TGF-β, and LOX-1 with the concomitant increase of EMT-inducing transcription factors and the decline of ERα gene expression [54]. Another interesting correlation has been reported between the different LOX-1 splice variants and different phenotypes of breast cancer [50]. The experiments were focused on the splice variant LOX-1Δ4 other than LOX-1. Authors have observed a different expression of LOX-1 isoforms depending on breast cancer phenotypes. This supports the hypothesis that the action exerted by LOX-1 splice variants is specific and related to individual molecular phenotypes, because particular activated pathways could impact on it [50]. LOX-1 and LOX-1Δ4 upregulation in vitro leads to a strong enhancement of proliferative rate and at the same time to a downregulation of cell-death-related proteins. The upregulation also stimulates a strong modulation of important factors of DNA double-strand breaks machinery, particularly embodied by Ku70 and histone H4 acetylation pattern [50]. In light of these evidences, LOX-1 could represent a target and prognostic factor for breast cancer, supplying a possible therapeutic option specific for different phenotypic cancer subtypes.

## LOX-1 and pancreatic cancer

Pancreatic cancer (PC) is the seventh leading cause of cancer-related deaths worldwide [55]. It represents one of the most lethal solid organ tumors, because its related symptoms remain silent until an advanced stage of the disease [55]. Globally, 458,918 new PC cases have been reported in 2018, with 432,242 of new deaths [55].

The treatment choice must be done in view of the stage of the disease at the diagnosis, and it can combine surgery, chemotherapy, chemoradiotherapy, and supportive care [56].

A study investigated the role of LOX-1 in PC starting from evidences about its involvement in cancer development and metastasis [57]. In particular, the authors proceed from studies highlighting its action in the increase of intracellular ROS levels and the consequent endothelial dysfunction. They first noticed an upregulation of LOX-1 in pancreatic tumoral cells derived from patients respect to their non-tumoral adjacent tissues. Furthermore, they established a correlation between LOX-1 expression and clinical pathological PC features. Specifically, a positive relationship between LOX-1 expression levels and the occurrence of lymph node metastases and high stage of the disease was determined. As prognostic factor, high LOX-1 expression can be considered an indicator of poor prognosis, also considering that its overexpression promotes the migration and invasion process of pancreatic cancer cells, but also the epithelial–mesenchymal transition [57].

Recently, a study has deepened the pathway involved in LOX-1-induced-PC metastasis [58]. The whole transcriptome RNA-sequencing analysis of pancreatic adenocarcinoma cells in which *OLR1* has been overexpressed or inhibited was performed. The outcome was the regulation of c-Myc and HMGA2 expression exerted by *OLR1* in order to promote pancreatic cancer metastasis [58]. In addition, *OLR1* seems to be involved also in chemoresistance of PC, mediated by the action of particular long noncoding RNAs, as GSTM3TV2. This lncRNA is able to promote gemcitabine-resistance in pancreatic cancer cells through a mechanism of upregulation of both *LAT2* (Linker for activation of T-cells family member 2) and *OLR1* [59].

Finally, all these evidences suggest the use of LOX-1 as prognostic factor but also as possible therapeutic target for pancreatic cancer.

## LOX-1, lung and squamous non-small lung cancer

Non-small cell lung cancer (NSCLC) accounts for 85% of all lung cancers, the most common cause of cancer-related mortality world widely [60]. Lung adenocarcinoma (LUAD) is the most diagnosed histological subtype of non-small cell lung cancer [61]. Due to the presence of metastatic disease at an early stage, the prognosis for patients with LUAD is generally poor, with average 5-year survival rates of <20% [60].

BMI has been shown as a risk factor for a variety of high-risk diseases including cancer [62]. Cancer cells must ensure sufficient energy influx to guarantee their survival and so they undergo substantial lipid metabolic reprogramming, which has been newly recognized as a hallmark of cancer diseases.

*Long Jiang* and collaborators have demonstrated that the analysis of combination of body mass index (BMI) and *OLR1* expression could classified patients (exactly 131) with Squamous NSCLC according to their progression-free survival (PFS) [62]. Authors established the potential in targeting lipid metabolic genes as a novel strategy in cancer treatment. Their work investigated the existence of TGF-β1-C/EBPδ-Slug-LOX-1 axis in lung adenocarcinoma representing an outstanding mediator between lipid metabolic regulators and EMT-inducing transcription factors in the regulation of cancer metastasis. C/EBP family are known to regulate cancer cell growth, proliferation, motility, and death in cancer cells. In particular, C/EBPδ, screened among different transcription factors pivotal for lipid metabolism, resulted in a downstream target of TGF-β1 mediating in this way TGF-β1-induced cancer metastasis. TGF-β1 is a potent EMT inducer that transactivates EMT-transcription factors and especially the snail, TWIST, and ZEB families, which in turn activates downstream metastatic target genes, such as LOX-1. It in turns mediates the pro-metastatic effects of slug in lung adenocarcinoma increasing ox-LDL uptake. LOX-1 has an E-box motif on its promoter that is bounded by slug, snail, and ZEB on their target genes, in order to orchestrate cancer progression. Meanwhile, LOX-1 and ox-LDL mediate the pro-metastatic effects of Slug in lung adenocarcinoma and Slug-induced cancer metastasis is significantly suppressed by LOX-1 neutralizing antibody [62].

This study offers theoretic basis for discovering novel therapeutic target in the treatment of lung adenocarcinoma.

## LOX-1 and gastric cancer

Gastric cancer (GC) is one of the most common malignant tumor in the digestive system worldwide; even if there has been a decline in the incidence of GC due to the efficient prevention and treatment of *H. pylori* infection, GC has the second-highest incidence and mortality rate of all cancers [63].

The only curative treatment for early-stage GC is an adequate surgical resection. Unfortunately, most GC patients are diagnosed with advanced stage, which need to be combined with systemic chemotherapy and often the overall survival (OS) remains low [63].

The analyses of two independent mRNA microarrays from GSE27342 database and the cancer genome atlas (TCGA) cohort have allowed the identification of a LOX-1 upregulation in about 100 tissues of gastric cancer. In addition, authors found an association between high LOX-1 expression and invasion depth, lymph node metastasis, tumor, node, metastasis (TNM), and overall survival [64].

In vitro LOX-1 promoted cell migration and invasion, enhancing EMT in GC cells by the activation of PI3K/AKT/GSK3β pathways. LOX-1, after binding ox-LDL, could increase several pro-angiogenic factors, such as VEGF that contributes to tumor growth, invasion, and metastasis [64]. Regarding VEGF family, VEGF-C is primarily expressed and secreted from cancer tissues, and acts as a strong stimulator able to promote lymph angiogenesis and lymph node metastasis [65, 66].

In a recent paper written by Caiqui Ma et al., elevated levels of ox-LDL in plasma of GC patients were measured by ELISA and associated both with high levels of VEGF-C expression and lymph angiogenesis. The mechanism works by activating the NF-Kb signaling pathway through LOX-1 receptor [67].

Thus, LOX-1 has been classified as an independent predictive factor of poor prognosis in patients. LOX-1 inhibition mediates ox-LDL activation and thus represents a potential therapeutic target for the prevention and intervention of early lymph node metastasis in gastric cancer.

## LOX-1, polymorphonuclear myeloid-derived suppressor cells, and glioblastoma multiforme

Polymorphonuclear myeloid-derived suppressor cells (PMN-MDSCs) are important regulators of immune responses in cancer and have been directly involved with the tumor progression [68, 69] PMN-MDSCs is phenotypically and morphologically similar to neutrophils (PMNs). Using partial enrichment of PMN-MDSCs with gradient centrifugation, it is possible to define low-density PMN-MDSC and high-density neutrophils [70]. Between these two groups, exist a big difference in terms of LOX-1 expression. In fact, while ox-LDL receptor is undetectable in neutrophils in peripheral blood of healthy donors, 5–15% of total neutrophils in cancer patients and 15–50% in tumor tissues were LOX-1 positive. In this study, it has been demonstrated that LOX-1 expression is not just associated to, but actually defines the population of PMN-MDSCs in cancer [71]. Then a combination of neutrophil markers with LOX-1 potentially allows for detection of PMN-MDSC in tissues. In fact, a dramatic increase in the number of PMN-MDSCs was observed in tumors of patients with head and neck cancer, colon cancer, non-small cell lung cancer, and multiple myeloma.

Glioblastoma multiforme (GBM) is the most common malignant primary brain tumor, with an incidence of 3.19 cases per 100,000 person-years [72]. A subset of PMN, constitutively expressing LOX-1, was identified in a GBM patient cohort [73]. Interestingly, a negative correlation of LOX-1 positive PMN with effector immune cells has been found in GBM patients. In addition, an accumulation of LOX-1 in their tissues have been correlated with the early recurrence and the disease progression.

In light of this finding, the authors concluded asserting that LOX-1 positive PMNs could be targeted in order to restore immune function in GBM patients and to improve the current therapeutic effectiveness of neoadiuvant anti-programmed death 1 (PD1) immunotherapy.

## LOX-1 and osteosarcoma

Osteosarcoma (OS) is one of the most aggressive malignancies in skeletal system with mortality rate worldwide. In pediatric bone disease, OS occupies about 5% of pediatric cancer [74].

Over the past 20 years, the high mortality is due to the tendency of osteosarcoma to develop metastases. Thus, identifying molecular and cellular mechanisms of metastases turns out to be fundamental, in order to dissect a multistep process that mediates the migration and invasion of osteosarcoma cells from primary to a distant site. Osteosarcoma exhibits EMT due to the modulation of Twist, snail, and Smads, defined as EMT-related transcription factors. These elements are involved in the complex invasive and metastatic behavior of osteosarcoma progression.

A tissue microarray analysis was performed and, among 24 genes found overexpressed in metastatic tumors, if compared to primary tumor genes, LOX-1 plays a central role in metastasis formation [75]. The overexpression of LOX-1 in both primary and metastatic OS tissues leads to a poor prognosis determining a malignant OS behavior in part via EMT approach.

Silencing *OLR1* gene in vitro in two OS cell lines (143b and MG63) overexpressing LOX-1, allows to inhibit osteosarcoma cell invasion and migration and to prevent in vivo metastasis formation. In addition, the silencing of *OLR1* repressed the expression of mesenchymal markers (snail, twist, and N-cadherin), but induced the epithelial marker E-cadherin. Integrally, the present study shows a novel step forward in comprehending the effect of this scavenger receptor in osteosarcoma metastases, evidencing a potential target for novel therapeutic approaches.

## Conclusion

This review provides an updating knowledge of the evidence acquired about LOX-1 involvement in tumor insurgence and progression in different organs, such as colorectal, breast, liver, pancreas, lung, brain, and bone. The ox-LDL receptor represents a fine-tuned interplay between lipid metabolic regulator, angiogenesis, and EMT-inducing transcription factor in the regulation of cancer metastasis. It has to be noted that *OLR1* is not always overexpressed in every cancer type nor in all the cell lines deriving from them, but, when functionally involved, it results to be overexpressed or abnormally spliced. In these conditions, it could represent a molecular therapeutic target for the inhibition of tumor progression and metastasis formation, allowing designing a personalized therapy. Unraveling the mechanisms underlying LOX-1 action will open up avenues to design new molecular treatment focused to inhibit its expression and indirectly angiogenesis process.

Remarkably, *OLR1* is critical in maintaining the transformation and growth states of cancer cell lines in diverse origins [45]. This phenomenon observed in xenografts experiments indicates the importance of LOX-1 in connecting cancer disease and metabolic disorder (https://www.who.int/cancer/prevention/diagnosis-screening/breast-cancer/en/). Studies also suggested multiple potential associations between *OLR1* and cancer susceptibility: *OLR1* overexpression in human cancer cells stimulates cell proliferation inducing tumor angiogenesis and demonstrating a direct relationship between obesity factors and the enhancement of proliferation and pro-angiogenic markers [36].

Moreover, LOX-1 expression could be used to monitor both the early detection of disease recurrence, and the “real-time” assessment of treatment effectiveness. Thus, it could represent a prognostic and diagnostic marker for pathological conditions such as cancer.

**Fig. 3:**
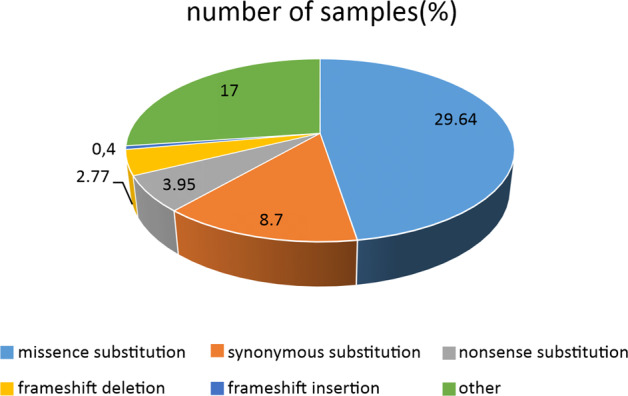
COSMIC data on somatic OLR1 mutations. A chart showing an overview of the different somatic mutations detected in *OLR1* gene.

Since in COSMIC (https://cancer.sanger.ac.uk/cosmic/gene/analysis) several *OLR1* somatic variants have been characterized (Fig. 3), a correlation between mutations in *OLR1* gene and the overexpression of its transcript or a dysregulated activity in cellular transformation has not been elucidated yet.

Importantly, early diagnosis and treatment of tumors can be optimized even when cancerous tissues are not available, measuring LOX-1 biomarker in liquid biopsy, as demonstrated in the serum of CRC patients.

Last, VOC analyses in vivo can be considered a non-invasive method for indicating the presence of compounds possibly modulated by LOX-1 expression and related to tumorigenesis. Such a technique may also allow non-invasive monitoring of response to therapy or revolutionize screening practices for some cancer types.
